# Use of the measure your medical outcome profile (MYMOP2) and W-BQ12 (Well-Being) outcomes measures to evaluate chiropractic treatment: an observational study

**DOI:** 10.1186/2045-709X-19-7

**Published:** 2011-03-20

**Authors:** Barbara I Polus, Amanda J Kimpton, Max J Walsh

**Affiliations:** 1 Division of Chiropractic, School of Health Sciences, RMIT University, Plenty Rd Bundoora, Melbourne, Australia

## Abstract

**Background:**

The objective was to assess the use of the Measure Yourself Medical Outcome Profile (MYMOP2) and W-BQ12 well-being questionnaire for measuring clinical change associated with a course of chiropractic treatment.

**Methods:**

Chiropractic care of the patients involved spinal manipulative therapy (SMT), mechanically assisted techniques, soft tissue therapy, and physiological therapeutic devices.

Outcome measures used were MYMOP2 and the Well-Being Questionnaire 12 (W-BQ12).

**Results:**

Statistical and clinical significant changes were demonstrated with W-BQ12 and MYMOP2.

**Conclusions:**

The study demonstrated that MYMOP2 was responsive to change and may be a useful instrument for assessing clinical changes among chiropractic patients who present with a variety of symptoms and clinical conditions.

## Background

In an era of accountability, health care providers are increasingly required to use reliable and valid outcome measures to assess changes in patient characteristics, including function and activities of daily living, following intervention. A review of outcome measures for primary care illustrates the evolution of instruments that acknowledge the importance of subjective perceptions of health and which focus on the measurement of function and quality of life [1].

Subjective outcome measures provide another dimension in the clinician's understanding of the patient's complaint when compared to standard objective measures (such as range of motion, palpation). Common subjective outcome measures include condition-specific tools such as the Revised Oswestry Disability Index and Neck Disability Index for assessing functional disability due to low back and neck pain respectively. Standardised questionnaires such as the Short form 36 (SF36) and the Well-being Questionnaire (W-BQ12) are used to assess general health status or quality of life - especially changes in self-concept over time following therapeutic intervention.

A recent approach is to assess change over time for specific symptoms or complaints identified by patients to be most important to them [1-3]. The Measure Yourself Medical Outcome Profile (MYMOP) has been recently developed to evaluate such patient-generated measures over time following therapeutic intervention [1]. The MYMOP is a brief patient generated, problem specific questionnaire which requires the respondent to specify one or two symptoms which are concerning them most and which they are seeking treatment for. A daily activity that is being restricted or prevented by these symptoms is also documented [4].

The MYMOP was initially published in 1996 [1] and was revised to MYMOP2 after a second validation in 1999 and included another section relating to medication [3]. It is a sensitive measure of within-person change over time; is capable of measuring the effects of a wide variety of care; and is a brief and simple questionnaire that can be completed during a consultation [1].

It has been used successfully to evaluate patient outcomes in a number of clinical settings including acupuncture [2,5], massage therapy in an Aboriginal community [6], acute exacerbations of chronic bronchitis [7], and more recently chiropractic management of patellar tendinopathy [8].

In the past the Short Form 36 (SF-36) has been the principal outcome measure for overall health in primary care. There are a number of studies that have evaluated the effectiveness of chiropractic care on patient's health and general health status as measured by the Short-Form 36 [9,10]. The MYMOP provides health practitioners with an alternative that is more easily incorporated into the practice setting because of its brevity. A comparative study of MYMOP and the SF-36 has been conducted [1]. MYMOP concurrent validity was supported by its ability to detect different degrees of change in relation to scores in acute and chronic conditions, and by its correlations with SF-36 scores. MYMOP correlated more closely with the subjective clinical findings than the SF 36. Paterson's study also showed that the MYMOP measure was capable of being responsive to changes in symptoms despite being brief.

The 12-item Well-being Questionnaire (W-BQ12) is another patient-centred subjective outcome measure that is geared towards people with long-term illness and has been found to be reliable and valid [11,12]. The W-BQ12 and MyMOP2 are two patient-centred outcome measures that are part of a set of five questionnaires that have been recently assembled to assess a range of outcomes experienced by people having acupuncture for long-term health problems [13].

Two of these five patient-centred survey instruments have recently been used to evaluate outcomes experienced by patients in response to body wall therapies such as massage [6] and chiropractic [8]. It was considered a significant step forwards to assess the utility of these questionnaires in another practice setting.

Therefore the aim of this observational study was to assess the utility of the MYMOP2 and W-BQ12 health outcomes measures for measuring clinical change associated with a course of chiropractic treatment delivered by student chiropractors in a clinical teaching facility. The W-BQ12 was also used as a tool to assess the validity of the well being component of the MyMOP2 against the validated W-BQ12 instrument in this clinical practice setting.

## Methods

A prospective, multicentre, practice based, observational study was conducted using patients presenting with spinal complaints to the RMIT University (Melbourne, Australia) chiropractic teaching clinics. For this observational study the patient's presenting complaint was not limited to a specific condition. Any patient who fulfilled the inclusion criteria was invited to participate in the study and were reviewed after 6 weekly treatments. The RMIT Human Research Ethics Committee approved all protocols and forms utilised for the study.

Patients were invited to participate in the study if they were: over the age of 18 years; had no treatment from any health professional for their complaint in the preceding four weeks; and suffered from a condition amenable to treatment by one or more chiropractic therapies. Patients were excluded if the following criteria were met: a requirement for immediate referral for medical treatment or where chiropractic intervention was contraindicated such as fracture, infection e.g. septic arthritis or malignancy; any additional physical treatment for their complaint during the course of the study; inability to complete or understand the required informed consent or outcome measures and inability to comply with the treatment schedule.

Under supervision of qualified chiropractic clinicians, treatment was provided by final year student chiropractors. Assessment prior to treatment included a full clinical history, physical, orthopaedic, neurological, palpatory and radiological examination. All participants received one or more chiropractic techniques taught and applied in the RMIT University chiropractic teaching clinics. These treatment protocols included: manual manipulative procedures such as spinal manipulative technique of high-velocity and low-amplitude thrust (SMT); soft tissue therapy; Logan Basic technique; and mechanical-force manually-assisted manipulation such as biomechanical blocking, drop-piece and activator. Segmental spinal dysfunction (subluxation) was assessed as described by Gatterman [14]. Patient management also included advice on nutrition, exercise and static stretching regimens as required.

### Outcome Measures

Two health and well-being questionnaires were used with consenting patients prior to and after completion of 6 treatments delivered over a minimum of one month and a maximum of three months. The questionnaires were either self-completed or administered by a student chiropractor if the patient requested this. The questionnaires were:

▪ 12 Item Well-being Questionnaire (W-BQ12)

**Figure 1:**
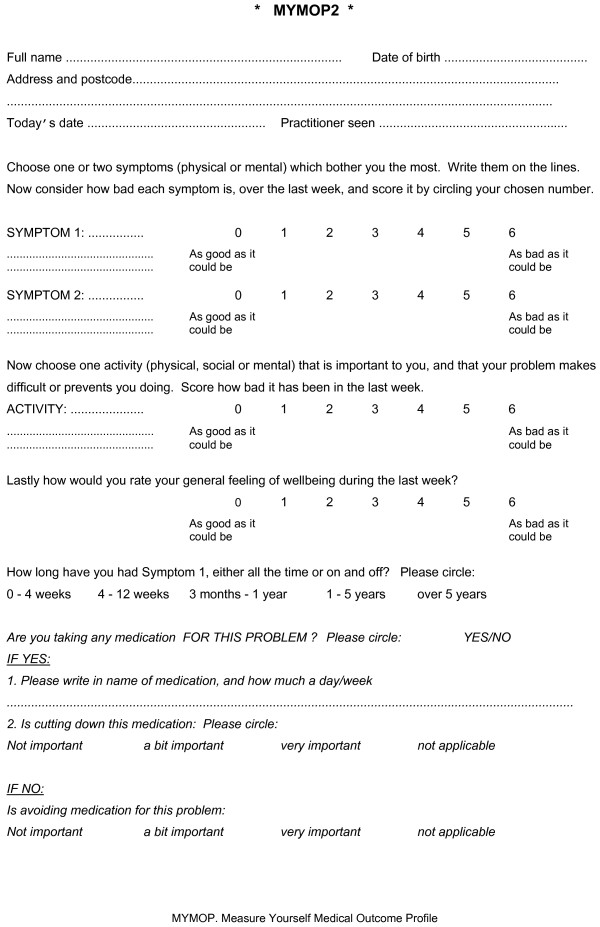
**MYMOP2 questionnaire**.

▪ Measure Yourself Medical Outcome Profile v2 (MYMOP2 - see Figure 1)

A description of the MYMOP2 subcategories is given in Table 1.

**Table 1:** Description of MYMOP2 subcategories

Category	Code	Description
**Symptom 1**	S1	The symptom which is most important to the patient described in the patient's own words.

**Symptom 2**	S2	Optional and is second symptom which is **part of the same problem **as symptom 1

**Activity**	A	An activity of daily living of importance to the patient in which Symptoms 1 and 2 interfere with. Written in patient's own words

**Well-being**	W	Patient asked how they would rate their general feeling of well-being over the last 7 days on a scale of 0 to 6, with 6 being as bad as it could be

**Profile**	P	Equals the mean of the scores recorded.

The W-BQ12 is a 12-item scale measuring four components: positive well-being (PWB), energy (E), negative well-being (NWB) and general well-being (GWB). Items 1-4 are summed to produce the negative well-being score; Items 5-8 produce a total energy score; and Items 9-12 produce the positive well-being score. The negative well-being score is reversed and then added with the energy and positive well-being scores to produce a general well-being score (range: 0-36). The higher the score on this reliable and valid instrument, the greater sense of general well-being [15].

The Measure Yourself Medical Outcome Profile [3] is a 'patient-centred' outcome scale where patients are asked to nominate one or two symptoms (physical or mental) of a specific problem they need assistance with and consider the severity of these symptoms over the last week. The third item asks the patient to list an activity (such as walking) that they have had difficulty completing due to their problem. The fourth item asks patients to rate their general well-being over the last week. Student chiropractors inserted the previously chosen symptoms and activity onto the follow-up form prior to this being given to the patient to score. Therefore, the patient was aware of the symptoms they had previously nominated, but not the previous score. Each of the four items is rated on a seven point scale where 0 is *'as good as it could be' *and 6 *'as bad as it could be'*. Hence, a decrease in the MYMOP2 score represents an improvement in health outcome. A mean of the four item scores is calculated and is referred to as the MYMOP2 "profile score".

The latest version of the MYMOP2 questionnaire (MYMOP2) was used in the present study and comprises another section relating to medication [3].

### Data analysis

All data were coded and entered into an Excel spreadsheet and then imported into SPSS v16.0 to perform statistical analysis.

The Wilcoxon signed rank test was used to compare baseline and post-treatment values for the outcome measures to investigate the responsiveness or sensitivity to change of both instruments.

Unpaired t-tests were used to compare the baseline (pre-treatment) characteristics of the group of patients who completed both initial and follow-up outcome measures and the initial total group. This test was completed to ensure that there was no difference in characteristics between the two groups (no follow-up and follow-up groups).

Chi-squared calculations were used to assess differences in pre-treatment categorical data.

Correlations between MYMOP2 and W-BQ12 scales were analysed using Spearman's correlation coefficients (r_s_) as a measure of the responsiveness, validity, in terms of well-being, and clinical usefulness of the instruments in a chiropractic student clinic setting.

All significance levels were set at p < .05.

## Results

Fifty-two (52) patients agreed to participate in the study, with each patient completing the MYMOP2 and W-BQ12 questionnaires prior to initial treatment.

Of the initial 52 subjects, 33 completed the full treatment schedule and were re-assessed after six treatments. There were no significant differences between the baseline (pre-treatment) characteristics of the total initial group (N = 52) compared to the group who completed the base-line and follow-up surveys (N = 33).

### Region of chief complaint

Back and/or neck pain was the most common presenting complaint, experienced by 71.2% of the initial sample of patients, with no significant differences between males and females in presenting region.

There was no significant difference in the distribution of region of main symptom between the total initial sample and the treatment group.

### Pre-treatment MYMOP2 scores

The MYMOP2 scores from the initial consultation are documented in Table 1. A MYMOP2 score of 6 represents 'as bad as it could be' and a score of 0 represents 'as good as it could be'.

While scores for females tended to be higher than for males for all sub-scores of the MYMOP2, there were no statistically significant differences except for profile scores where females had a statistically significantly higher score (p = .004).

### Age groups

**Figure 2:**
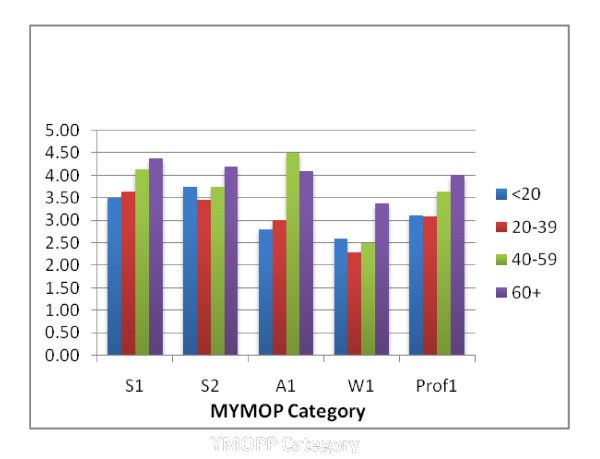
**Presenting mean MYMOP2 scores according to age group**.

The distribution of presenting (pre-treatment) MYMOP2 scores according to age groups is shown in Figure 2.

The 52 subjects were broken down into the following age groups: <20yo (n = 5), 20-39 (n = 25), 40-59 (n = 15) and >60 (n = 7).

The older age groups tended to have higher scores across each sub-score but there were no significant differences between the various age groups.

### Treatment effects on MYMOP2 and W-BQ12 scores

**Figure 3:**
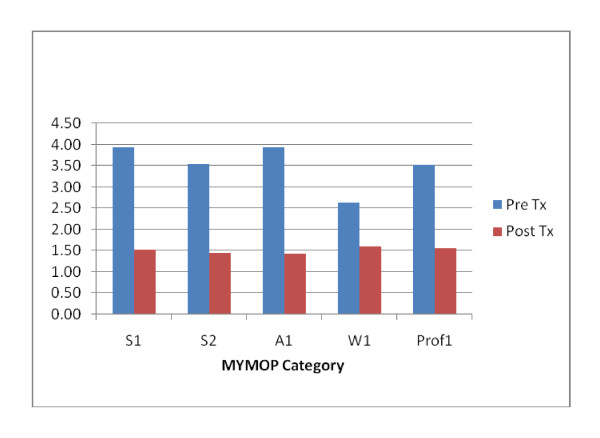
**Comparison of pre and post treatment MYMOP2 scores**.

**Figure 4:**
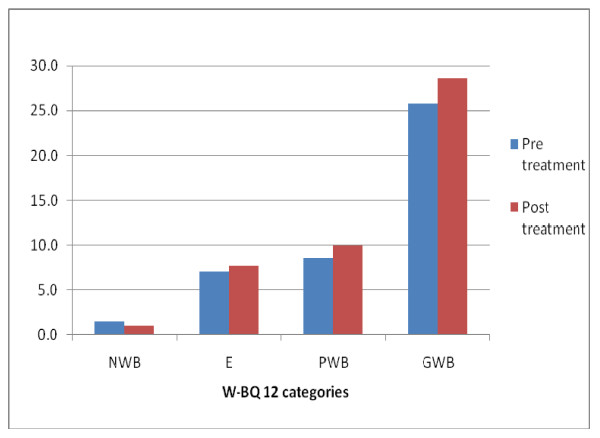
**Comparison of pre and post treatment WBQ-12 scores**.

The effect of treatment on MYMOP2 and W-BQ12 scores is shown in Table 2 and Figures 3 and 4 respectively. Large significant changes occurred in all MYMOP2 categories following treatment (p < .0001), with improvements over baseline from 40 to 65 percent.

**Table 2:** Patient characteristics

	Total group (n = 52) Pre-Tx data	Tx group (n = 33) Pre-Tx data	Tx group (n = 33) Post-Tx data
**Gender**			
**Male**	24 (46.1%)	16 (48.5%	
**Female**	28 (53.9%)	17 (51.5%)	

**Age Mean -yrs (SD)**	39.4 (17.3)	40.9 (18.4)	
**Range yrs**	18 - 82	18-82	

**Age categories -yrs**			
**<20**	5 (9.6%)	3 (9.1%)	
**20-39**	24 (46.1%)	14 (42.4%)	
**40-59**	15 (28.8%)	10 (30.3%)	
**<60**	8 (15.4%)	6 (18.2%)	

**Mean MYMOP2 scores (SD)^a^**			
**Symptom 1 S1 (SD)**	3.9 (1.1)	3.9 (1.1)	**1.5 (1.2)**
**Symptom 2 S2 (SD)**	3.6 (1.2)	3.5 (1.2)	**1.5 (1.1)**
**Activity A (SD)**	3.9 (1.4)	3.9 (1.5)	**1.4 (1.1)**
**Well-being W (SD)**	2.5 (1.3)	2.6 (1.3)	**1.6 (1.3)**
**Profile P (SD)**	3.4 (1.0)	3.5 (0.9)	**1.6 (1.0)**

**Mean W-BQ12 scores (SD) ^b^**	**1.4 (1.5)**	**1.4 (1.3)**	**1.0 (1.3)**
**Negative well-being NWB Energy E**	**7.1 (2.5)**	**6.9 (2.5)**	**7.7 (2.5)**
**Positive well-being PWB**	**8.6 (2.5)**	**8.5 (4.4)**	**10.0 (4.4)**
**General well-being GWB**	**26.1 (5.4)**	**25.8 (5.8)**	**28.6 ()**

SD = standard deviation

**^a ^**MYMOP2, 6 is "as bad as it can be" and 0 is "as good as it can be"

^b ^W-BQ 12,each subscale has a maximum score of 12 except total well-being score which has a maximum of 36.

The W-BQ-12 scores were negative well-being (NWB), Energy (E), Positive Well-being (PWB) and General Well-being (GWB). Figure 4 compares the pre treatment and post treatment scores. All W-BQ12 scores showed a significant improvement in scores following treatment (p < .05), noting that a decrease in negative well-being corresponds to a positive effect of treatment.

### Correlation between MYMOP2 and W-BQ12 scores

Correlations between MYMOP2 scales and W-BQ12 scales were assessed using Spearman's correlation coefficients (r_s_) as shown in Table 3.

**Table 3:** Correlation coefficients for MYMOP2 vs W-BQ12 scales

	W-BQ12 scales	General Well-Being	Positive Well-Being	Energy	Negative Well-being
**MYMOP2 Scales**					

**Symptom 1** **(S1)**	r_s_ p (2-tailed)	- .330 .015	-.221* .107	.320 .018	.058* .675

**Activity (A)**	r_s_ p (2-tailed)	-.229* .103	-.037* .792	-.268* .058	.058* .682

**Wellbeing (WB)**	r_s_ p (2-tailed)	- .512 < .001	-.311 .022	-.445 .001	**.358** **.008**

**Profile (P)**	**r_s_** **p (2-tailed)**	**-.372** **.006**	-.172* .212	**-.370** **.006**	.201* .144

* = no statistical significance

The MYMOP2 scales of Symptom 1 and Profile showed a moderate negative correlation with the General Wellbeing (GWB) and Energy scales of the W-BQ12. The Wellbeing scale of the MYMOP2 had a strong negative correlation with the GWB, a moderate negative correlation with the PWB and Energy scales and a positive moderate correlation with the Negative wellbeing scale.

The Activity scale of the MYMOP2 had no significant correlations with any of the W-BQ12 scales.

Correlations between MYMOP2 scales and W-BQ12 scales were assessed using Spearman's correlation coefficients (r_s_) as shown in Table 3.

## Discussion

This observational study had two objectives. The first objective was to assess the effectiveness of the MYMOP2 and W-BQ12 questionnaires in measuring clinical changes following chiropractic care on patients attending the RMIT University chiropractic teaching clinics. The second objective was to investigate the validity of the MyMOP2 instrument to detect a change in well-being of patients attending the RMIT chiropractic teaching clinic.

The mean baseline MYMOP2 profile score was 3.4 (+/- 1.0) for the 52 presenting chiropractic patients as demonstrated in this study which is similar to that obtained in a study of massage therapy for subjects with chronic musculoskeletal complaints [6]. It is lower than those of patients attending for acupuncture in medical practices (4.7) [2], and for those patients attending general practice in the UK (4.6) [1]. The presenting MYMOP2 scores were not dependent on age or gender except for the Profile sub score where females had a significantly higher score. Given there is no difference in other sub scores there is no apparent reason why females should have a higher Profile score.

There was a statistically significant improvement in all MYMOP2 sub-scales following chiropractic treatment indicating a positive effect of the therapy. These changes were similar to changes found in the other studies referred to above.

The improvements were also of clinical significance defined as a change in score that is of importance to the individual patient involved. The MYMOP2 uses a 7-point score for which the minimum clinically important change in score after intervention should be between 0.5-1.0: any change greater than 1.0 can be considered clinically significant [16].

The changes in all MYMOP2 scores were equal to or greater than 1.0 (for Symptom 1 and Symptom 2 changes were greater than 2.0), suggesting that, in general, the effect of therapy was clinically significant to patients.

There were also significant improvements in the W-BQ12 scores, once again suggesting a positive effect of the treatment. According to Pouwer et al [15], the W-BQ12 is a reliable and valid measure of well-being and has been used in a number of studies to measure clinical changes following treatment [6,17,18]. It is of interest to compare the changes observed in the W-BQ12 in our study with that of another recent large study that measured a range of treatment effects of traditional acupuncture - including changes in self concept - the target of the W-BQ12 [19]. In this latter setting, the W-BQ12 was not found to be responsive. The authors of this latter study attributed the lack of responsiveness of the W-QB12 to two possible causes: either the socioeconomically diverse population or the preponderance of musculoskeletal problems present in their sample. While our study is unable to comment on the first possibility, all participants in our study presented with musculoskeletal pain of spinal origin. Therefore, in contrast to the Paterson *et al *study [19], our study suggests that the W-BQ12 may be a useful outcome measure for use within a chiropractic clinical practice setting.

The correlation between MYMOP2 and W-BQ12 scores was moderate to strong for most scales other than the Activity scale of the MYMOP2 which had no significant correlations with any of the W-BQ12 scales (see Table 3).

MYMOP2 has been shown to be highly responsive to changes in symptoms whether acute or chronic, as well as correlating with the findings of the SF-36 [1].

Based on this and the observational findings of this study, the MYMOP2 has potential as a clinically useful tool to assess chiropractic care in terms of health status and general well-being. The official MYMOP website [16] lists the strengths and weaknesses of the MYMOP2 questionnaire. The major strengths are considered as: patient-centred, applicable to any problem, quick and easy to complete and score, and very responsive to change. The main weakness is that it is problem specific which makes it unsuitable for patients who cannot identify their problem.

The use of a non-experimental (observational) study design has well-established limitations. First, it is not possible to attribute any change to the intervention itself as other confounding effects (notably natural history and regression to the mean), could be responsible for the change observed. However, as the changes observed were both statistically and clinically significant, such an interpretation is less likely. Further, the purpose of the study was to document how these patient-centred outcome measures performed in a chiropractic clinical practice setting. A non-experimental, observational research design was considered appropriate for such an investigation and minimised disruption to the provision of the chiropractic service.

Another limitation of this observational study was that the practitioners were student chiropractors with minimal clinical experience. This may have had some impact on the observed findings as well as influencing the external validity of the study.

## Conclusions

This study assesses the use of the MYMOP2 and W-BQ12 questionnaires as outcome measures to monitor changes following chiropractic therapy. Within the limitations of this study, it was shown that both questionnaires were responsive to change. The MYMOP2 also correlated well with the W-BQ12 questionnaire. It thus appears to be a useful instrument for assessing change among chiropractic patients and in the assessment of patient perceived well-being for chiropractic patients who present with a variety of symptoms and clinical conditions.

## Competing interests

The authors declare that they have no competing interests.

## Authors' contributions

BP conceived the study, participated in its design and its coordination. BP, AK and MW supervised the student chiropractors in the collection and analysis of data. MJW undertook a further overall statistical analysis of data and drafted the manuscript. All authors read and approved the final manuscript.
